# Do dysfunctional metacognitive beliefs contribute to interpersonal distress beyond interpersonal styles, parental bonds, depression and anxiety? A prospective within-person study

**DOI:** 10.3389/fpsyt.2026.1766358

**Published:** 2026-02-24

**Authors:** Eivind R. Strand, Frederick Anyan, Henrik Nordahl

**Affiliations:** 1 Department of Child and Adolescent Mental Health, Sorlandet Hospital, Kristiansand, Norway; 2 Department of Psychology, Norwegian University of Science and Technology, Trondheim, Norway

**Keywords:** interpersonal problems, interpersonal style, longitudinal, metacognition, S-REF model, within-person analysis

## Abstract

**Background:**

General interpersonal problems and distress are transdiagnostic features across the psychopathology spectrum and relate to reduced quality of life, greater emotional symptom severity, and worse outcomes following psychotherapy. Identifying within-person dynamic factors influencing interpersonal distress could therefore advance clinical formulation and intervention, benefiting a large number of patients.

**Methods:**

Based on a four-wave longitudinal study, we used latent growth modelling to investigate whether metacognitive beliefs, the key mechanism of psychological dysfunction in the metacognitive model, predicted the trajectory of interpersonal distress within individuals over time. We controlled gender, parental bonds to mother and father, and interpersonal style factors agency and communion at baseline, in addition to time-varying changes in anxiety and depression symptoms at the within-person level.

**Results:**

We found that all the predictors except for gender were associated with greater interpersonal distress at baseline on the between-person level. Between-person differences in parental bonds and interpersonal style did not predict the trajectory of interpersonal distress over time. Increases in anxiety and depression symptoms at the within-person level predicted greater interpersonal distress within individuals over time. Finally, all metacognitive belief domains assessed with the MCQ-30 except cognitive self-consciousness were unique predictors of greater interpersonal distress over time within individuals beyond the included between- and within-person covariates.

**Conclusion:**

These findings suggest that targeting metacognitions could be relevant to alleviate interpersonal distress, potentially independent of parental bonds, interpersonal style configuration and within-person fluctuations in emotional disorder symptoms.

## 1 Introduction

Interpersonal problems can be described as persistent difficulties individuals experience in their social interactions and relationships causing substantial distress for the individual but often for others close to them as well (1). They are more pronounced in clinical than non-clinical populations (2) and prevalent across psychiatric disorders, including depressive- (3), anxiety- (4, 5), and personality disorders (6).

Although some diagnostic groups display signature interpersonal styles (7, 8), research more often indicates marked heterogeneity of interpersonal problems within diagnostic categories (6, 9). Consequently, general interpersonal problems and distress can be viewed as transdiagnostic features of psychopathology, and they are linked to greater emotional symptom severity (e.g., 10, 11) as well as poorer social, occupational, and quality-of-life outcomes (12, 13). In addition, general interpersonal problems are associated with worse outcomes following psychotherapy (14). Therefore, illuminating factors underlying interpersonal problems and distress with direct clinical implications for conceptualization and interventions is of high relevance to many patients across the psychopathology spectrum.

Several factors have been hypothesized as relevant for understanding interpersonal problems and distress. A common notion is that they might be formed and related to early attachment experiences in close relationships suggested as a foundation for more stable cognitive internal working models of self and others (e.g., 15, 16). Although not equivalent to comprehensive assessments of attachment styles, self-reported parental bonds to mother and father based on the care received the first 16 years of life have been associated with and suggested as causally related to an increased risk of psychopathology and interpersonal functioning later in life (17). Other perspectives highlight the role of trait-level factors such as maladaptive personality dispositions as central vulnerability factors contributing to resulting interpersonal distress and functioning over time (18). Interpersonal styles characterized by detachment have been associated with interpersonal problems and psychopathology risk more broadly (e.g., 19). Interpersonal problems and distress have further been found to co-vary with symptoms of anxiety and depression (e.g. 10, 20, 21) indicating that they may reciprocally influence each other. Finally, factors such as gender could also play a role given that some studies have found general interpersonal problems and specific stylistic features to vary across genders (22).

The Self-Regulatory Executive Function (S-REF) model (23, 24), often referred to as the metacognitive model of psychological disorders, proposes that the commonalities across disorders are more important than the differences. In this perspective, psychological disorders and dysfunction such as interpersonal problems and distress are related to underlying biases within the *metacognitive control system* (MCS; 23). Biases in the MCS (e.g., metacognitive knowledge about lack of cognitive control) are thought to negatively influence self-regulation strategies. Unhelpful self-regulation strategies which are under the influence of biases in metacognition are collectively called the *Cognitive Attentional Syndrome* (CAS; 25). The CAS comprises perseverative negative thinking (e.g., rumination/worry/self-criticism), threat-monitoring in the form of strategic and danger-oriented focus of attention, and maladaptive coping behaviours (e.g., self-harm, avoidance, excessive reassurance-seeking). In this perspective, interpersonal problems can be part of the CAS given that interpersonal behavioral strategies such as being overly domineering or self-sacrificing are classified as top-down influenced strategic behaviours. Additionally, interpersonal distress can also result from CAS strategies. For example, worrying or self-criticism can disturb effective communication (e.g., reciprocity or empathy) and impair self-esteem and assertiveness which may *contaminate* social interaction and relationships. Hence, the S-REF conceptualization of interpersonal problems and distress is that they are linked to underlying biases in metacognition.

There is now quite convincing empirical evidence for an association between metacognitive beliefs and interpersonal problems/distress. Dysfunctional metacognitive belief domains (i.e., positive beliefs, negative beliefs, cognitive confidence, and cognitive self-consciousness) were uniquely associated with general interpersonal problems in a non-clinical sample even when controlling attachment styles, emotional distress, and personality traits (26). In clinical samples, interpersonal problems are associated with biased metacognitive beliefs beyond negative cognitions in patients with social anxiety disorder (SAD) (27), and with meta-worry beyond trait-worry in patients with generalized anxiety disorder (GAD) (28). In patients with personality disorders (PDs) (where interpersonal problems are core diagnostic features), metacognitive beliefs are significantly elevated compared to patients without PDs (29). Furthermore, a recent systematic review and meta-analysis (30) reported that Metacognitive therapy (MCT; 25), which directly modify biases in metacognition, are associated with reduced interpersonal problems across diagnostic groups indicated by a large pooled effect size (*g* = 0.865, 95% CI [0.512–1.218]). Additionally, a recent study on the attention training technique (a metacognitive change technique used in MCT) applied to patients with mixed anxiety disorders, reported a positive and moderate to large effect on interpersonal problems (31).

Although previous studies have supported an association between metacognition and interpersonal distress, these findings have mainly been based on cross-sectional, correlational and between-subjects effects. The within-person level of analysis provides a more robust empirical test of the proposed association in line with the underlying S-REF theory and further provides some natural control concerning both stable attributes and spontaneous fluctuations at the individual level (e.g., general intelligence and physical health) (32). Additionally, we need more knowledge about the potential contribution of specific metacognitive belief domains as it may bring more precise implications for clinical practice.

In the current study, we therefore aimed to add to the previous literature by testing whether metacognitive belief domains independently predict interpersonal distress longitudinally and at the within-person level. We controlled empirically relevant factors such as gender (22) and perceived parental bonds to mother and father based on the care received the first 16 years of life at baseline (e.g., 33, 34). Furthermore, we controlled participants initial levels in two interpersonal style factors: *agency* related to autonomy, control, dominance or opposite submissiveness, and *communion* related to seeking closeness, affection, cooperation or opposite detachment (e.g., 19, 35). These factors are the central hypothesized stable style factors contributing to interpersonal stress over time according to contemporary interpersonal theory (e.g., 36, 37) and were therefore included as time-invariant factors at baseline. Finally, we included time-varying changes in symptoms of depression and anxiety at the within-person level given that interpersonal problems/distress have been found as reciprocally related to them (e.g. 20, 21) and changes in general interpersonal distress could therefore mainly be influenced by fluctuations in emotional symptom levels. In sum, these covariates contribute to a stringent test of the potential predictive role of metacognitive belief domains to general interpersonal distress at the within-person level over time.

We hypothesized that: 1) all predictors as outlined above would be positively associated with greater interpersonal distress at baseline (intercept), and of the trajectory over time at the between-person level; and 2) that all dysfunctional metacognitive belief domains would contribute above and beyond the included baseline characteristics and time-varying emotional disorder symptoms (anxiety and depression) in explaining greater interpersonal distress over time at the within-person level.

## 2 Methods

### 2.1 Participants and procedure

Participants over the age of 18 were recruited at convenience to take part in an online survey advertised through social media platforms. The same measures were administered 4 times separated by 5 weeks intervals. Participants had to be 18 years or above and able to read Norwegian in order to participate, but no other exclusion criteria were applied. Informed consent was provided before entering the survey. The Regional Committees for Medical and Health Research Ethics approved the study (Ref nr: 467342) and it was registered with the Norwegian Centre for Research data (Ref nr: 686857). In total, the sample at time 1 comprised 1418 individuals of which 669 (47.2%) were men, 728 (51.3%) women, 14 (1.0%) identified as non-binary, 4 (0.3%) answered that these categories could not describe how they identified, and 3 (0.2%) provided no information. The mean age was 29.75 years (*SD* = 11.67, range = 18-79). In terms of relationship status and education, a total of 753 (53.1%) were in a relationship, cohabitants or married, and 774 (54.5%) had a university degree of 3 years or more as their highest completed education. Regarding mental health problems, 478 (33.7%) reported having been diagnosed with a mental disorder during their lifetime.

### 2.2 Measures

The Inventory of Interpersonal Problems (IIP-32; 38) assesses difficulties people experience in their interpersonal relationships and is based on the version by Horowitz et al. (1) originally consisting of 127 items. Items are rated on a 5-point scale ranging from 0 (*not at all*) to 4 (*very or extremely*) and can be divided between eight subscales comprising unique interpersonal problem domains: being domineering, vindictive, cold, socially avoidant, nonassertive, exploitable, overly nurturant and intrusive. These can be portrayed in an interpersonal circumplex depicting interpersonal problems along the dimensions of agency (e.g., behaviors related to independence and autonomy, control, and assertiveness) vertically, and communion horizontally (e.g., behaviors related to closeness, affection and cooperation). When represented circularly, the eight subdomains are visualized as octants thought to represent constellations of the underlying orthogonal dimensions of agency and communion. High levels of agency thus represent an overly assertive/domineering style, while low levels a submissive/avoidant style. High levels of communion are associated with an overly warm/self-sacrificing style, while low levels are associated with a detached/cold interpersonal style. Additionally, a third factor capturing the general level of interpersonal distress irrespective of style (39) can be computed by an individual’s mean score across all subscales. In the current study we employed a dimensional scoring approach in line with recommendations by Wendt et al. (35) computing both the general interpersonal distress factor, in addition to interpersonal style factors (i.e., agency and communion). This is in line with previous studies who have used a similar approach (e.g., 40, 41). The internal consistency of the total scale in the current study was good (*α* = .90).

A shortened version of the Parental Bonding Instrument (PBI) by Parker et al. (34) especially developed for epidemiological research (42) measuring perceived parental care up to age 16 (mother and father evaluated separately) was utilized. The measure entails 16 items scored on a 4-point Likert scale ranging from 1 (“a lot” to 4 “not at all”). In the current study, the total score for fathers and mothers was used with items reverse coded to ensure that higher scores reflect suboptimal parental bonding (perceived lack of care, autonomy, and overprotection). The measure has demonstrated good psychometric properties including internal consistency for both the mother (α = .88) and father (α = .88) scale (43) In the current sample, the internal consistency was good for the mother (*α* = .90) and father (*α* = .88) scale.

The Generalized Anxiety Disorder 7 (GAD-7; 44) measures symptoms of generalized anxiety (e.g., feeling nervous, anxious or trouble relaxing) and was utilized in the current study to control for levels of anxiety symptoms. In total, 7-items are rated on a 4-point scale from 0 (“not at all”) to 4 (“nearly every day”). It has demonstrated excellent internal consistency (*α* = .92; 44), and in the current study the internal consistency was good (*α* = .89).

The Patient Health Questionnaire 9 (PHQ-9; 45) measures the nine criteria for depression specified in DSM-IV (e.g., feeling down, depressed, or hopeless) on a scale from 0 (“not at all”) to 3 (“nearly every day”), and was utilized in the current study to control for levels of depressive symptoms. It has been supported as a valid instrument for measuring depression (46) with good internal consistency (*α* = .89; 45). In the current study, the internal consistency was good (*α* = .90).

The Metacognitions Questionnaire 30 (MCQ-30; 47) measure five domains of dysfunctional metacognitive beliefs; positive beliefs (e.g., “Worrying helps me avoid problems in the future”), negative beliefs (e.g., “When I start worrying, I cannot stop”), cognitive confidence (e.g., “I have a poor memory”), need for control (e.g., “Not being able to control my thoughts is a sign of weakness”), and cognitive self-consciousness (e.g., “I monitor my thoughts). It has demonstrated good psychometric properties with internal consistencies ranging from.72 to 93. for the subscales (47). In the current study, the internal consistencies for the subscales were acceptable to good: *α* = .84 for positive beliefs; *α* = .87 for negative beliefs; *α* = .89 for cognitive confidence; *α* = .76 for need for control; and *α* = .81 for cognitive self-consciousness.

### 2.3 Statistical analyses

Systematic analyses of missing data patterns and trajectory plots were examined prior to the main analyses. Statistical analyses were performed in Mplus 8.10 (48) with Full-information Maximum Likelihood (FIML) method and robust estimation (MLR). A well-fitting latent growth curve model (LGCM) was employed to assess the overall trajectory of general interpersonal distress, capturing both the initial level and rate of change through two latent variables: *the intercept and slope growth factors*. Although alternative longitudinal SEM approaches such as the Random Intercept Cross-Lagged Panel Model (49) are well suited for testing prospective within-person directional effects, the present analyses were guided by a theoretical emphasis on modeling systematic change in interpersonal distress over time and concurrent within-person covariation with metacognitive beliefs, anxiety and depression symptoms. Several time invariant covariates including gender, parental bond to father and mother, interpersonal styles represented by agency and communion, were included in the growth model to explain between-person differences in the initial status and rate of change. Next, the time-varying effect of dysfunctional metacognitive beliefs (i.e., positive beliefs, negative beliefs, cognitive confidence, need for control, and cognitive self-consciousness) as well as anxiety and depression symptoms were included as dynamic predictors of the variation in general interpersonal distress over time. This was to test how fluctuations in the time varying covariates at each timepoint relate to fluctuations in general interpersonal distress at that same timepoint. In this framework, growth factors and known baseline time-invariant covariates represent level 2 effects (or between-person differences) while the exogenous time-varying effect of dysfunctional metacognitive beliefs and symptoms of depression and anxiety represent level 1 effects (or time level variations). The following fit indices determined adequate fit: Standardized Root Mean Square Residual (SRMSR <.08), Root Mean Square Error of Approximation (RMSEA ≤.06) (50) Comparative Fit Index (CFI ≥.90) and a non-Normed Fit index (NNFI; aka TLI ≥.90) (51).

## 3 Results

### 3.1 Analyses of missing data patterns

Attrition and missing data were examined prior to model estimation. The number of participants providing data at each wave was as follows: (Wave 1: *N* = 1418, Wave 2: *N* = 765, Wave 3: *N* = 650 and Wave 4: *N* = 611). The number of missingness for all variables included in the model (ranging from 0.28% to 57.0%) are contained in the **Supplementary Material**, **Supplementary Table S1**. Item-level missingness within completed surveys were less than (<58%). To assess the plausibility of random missingness participants who completed all waves were compared with those who did not on key demographic variables and baseline levels of the study variables. A series of Chi-square tests and independent *t*-tests were conducted to evaluate differences between study completers and non-completers across various demographic and the covariates. Statistical analysis revealed significant associations between completion status and relationship status *χ^2^* (4) = 18.614, *p* <.001, diagnosis *χ^2^* (1) = 8.896, *p* = .003, and sex *χ^2^* (1) = 9.749, *p* = .021, whereas education level showed no significant relationship (*p* = .190). Regarding baseline covariates, completers reported significantly higher initial depression symptoms (*t* = -2.438, *p* = .015) and were significantly older (*M* = 31.52) than non-completers (*M* = 28.40, *p* <.001). Additionally, a significant difference was found for agency (*t* = 4.29, *p* <.001), while no significant differences were observed for metacognitive beliefs, anxiety symptoms, or parental bonding (**Supplementary Material**, **Supplementary Table S2**).

For our primary outcome variable, covariance coverage across waves ranged from 35% to 99% (**Supplementary Material**, **Supplementary Table S3**). Additional missing data analyses on the primary outcome revealed that although attrition was not systematically related to interpersonal distress at earlier timepoints, by the final measurement completers reported slightly higher distress than non-completers (**Supplementary Material**, **Supplementary Table S4**). This suggests that participants reporting greater distress may have been more likely to remain in the survey. We also used logistic regression to estimate the extent to which variables in previous times (i.e., T1 – T3) predict attrition from subsequent times (T2 – T4). If variables in the analysis model are related to attrition, it is unlikely that dropout occurred completely at random (i.e., resulting in data that are not missing completely at random, MCAR). The results indicated that the logistic regression models were not significant in the extent to which variables in previous times predict attrition from subsequent ones (**Supplementary Material**, **Supplementary Table S5**). Finally, Little’s Missing Completely at Random (MCAR) test supported missingness at random: *χ^2^* = 19.79, *df* = 18, *p* = .344. These results, except the difference between completers and non-completers in the fourth measurement, support random missingness in the data, and the plausibility of FIML. FIML is regarded as a state-of-the-art missing data technique because it improves the accuracy and the power of the analyses relative to other missing data handling methods (52).

### 3.2 Preliminary results

The means and standard deviations (T1: Mean = 37.44, *SD* = 17.95; T2: Mean = 37.51, *SD* = 18.99; T3: Mean = 35.76, *SD* = 19.24; T4: Mean = 35.40, *SD* = 20.24) showed a pattern with decreases in general interpersonal distress over time, coupled with increases in variation. The correlations over time also present a simple pattern with most correlations suggesting a relatively high level of stability in individual differences (e.g., *r* >.84, *p* <.001). **Supplementary Figure S1** in the **Supplementary Material** is a display of observed individual trajectory plots for a random sub-sample (*n* = 50) of completers.

### 3.3 A growth model for the trajectory of general interpersonal distress

When examining various growth functions to model the trajectory of general interpersonal distress, the linear growth model (*χ^2^* = 12.98, *df* = 5; *p* <.05; *SRMR* = .01; *RMSEA* = .03; *CFI* = .99; *TLI* = .99) demonstrated a better fit to the data than the no-growth model (*χ^2^* = 124.26, *df* = 8; *p* <.00; *SRMR* = .10; *RMSEA* = .10; *CFI* = .93; *TLI* = .95). When compared to the linear growth model, the quadratic growth model (*χ^2^* = 5.21, *df* = 1; *p* <.05; *SRMR* = .01; *RMSEA* = .06; *CFI* = .99; *TLI* = .98) did not show a significant improvement in model fit (*χ^2^* = 8.07, *df* = 4; *p = .*09). Therefore, the linear growth model was selected for its greater parsimony. The intercept (*b* = 37.62, *p* <.001) and slope growth factors (*b* = -1.09, *p* <.01) were all significant with significant variance estimates (276.91, *p* <.001) and (3.93, *p* <.01), indicating significant individual differences in the initial level and the declining rate of change over time in general interpersonal distress. The *R^2^* values (i.e., explained variance) ranged between 84.90% and 93.60%.

### 3.4 Conditional growth models with time-invariant and time-varying covariates

**Figure 1:**
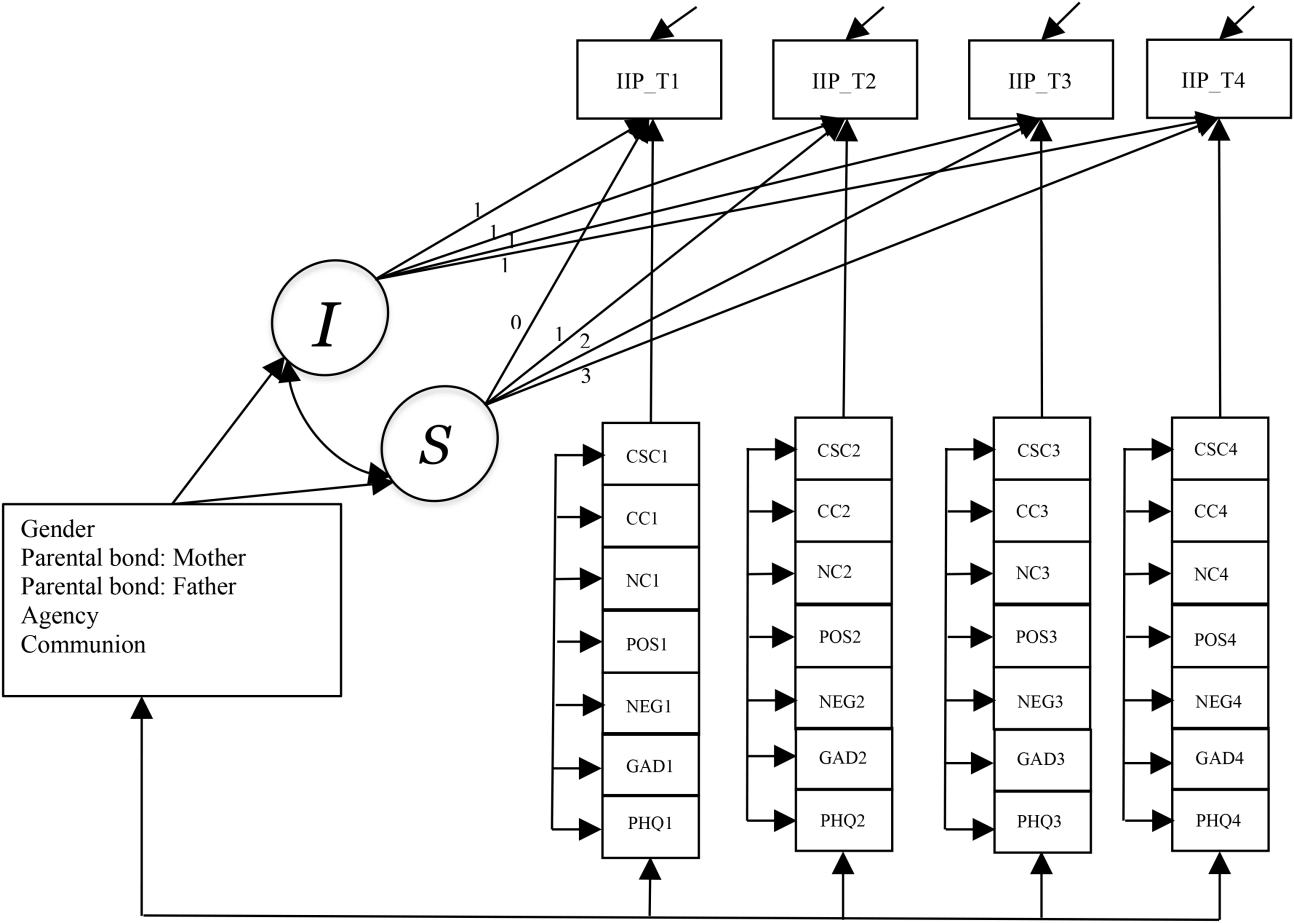
Latent growth curve model with time-invariant and time-varying covariates. I, Intercept growth factor; S, Slope growth factor; IIP, General interpersonal distress; NEG, Negative beliefs about the uncontrollability and danger of worry; POS, Positive beliefs about worry; NC, Need to control thoughts; CC, Lack of cognitive confidence; CSC, Cognitive self-consciousness; GAD, Anxiety symptoms; PHQ, Depression symptoms.

The model with the baseline time-invariant covariates (LGCM-TIC) showed an adequate fit to the data (*χ^2^* = 27.33, *df* = 15; *p <*.05; *SRMR* = .01; *RMSEA* = .02; *CFI* = .99; *TLI* = .99), and as did the model including the time-varying covariates (LGCM-TIC and TVC: *χ^2^* = 202.39, *df* = 99; *p = .*05; *SRMR* = .01; *RMSEA* = .01; *CFI* = .99; *TLI* = .99). In the final model, the time varying effects for each covariate was constrained equal over time, but there was no significant change in the model fit (*χ^2^* = 25.15, *df* = 21; *p = .*24). Parameter estimates for both LGCM – TIC, and LGCM – TIC and TVC models are shown in **Table 1**. **Figure 1** shows the final LGCM – TIC and TVC model.

**Table 1:** Parameter estimates for LGCM with time-invariant and time varying covariate models.

	Time-invariant covariate model			Time-invariant with time varying covariate model		
	*Est*	*S. E*	*p*	*Est*	*S. E*	*p*
Intercept	5.44	1.92	.005	5.97	1.81	.001
Slope	-2.37	0.61	.000	-1.94	0.59	.001
Intercept variance	167.73	9.90	.000	103.17	6.79	.000
Slope variance	4.01	1.28	.002	4.87	1.15	.000
Intercept with Slope	7.66	2.58	.003	1.66	2.22	.455
Intercept predicted by						
Gender	1.26	0.78	.105	-0.34	0.62	.578
Father bond	0.46	0.06	.000	0.34	0.05	.000
Mother bond	0.35	0.05	.000	0.19	0.04	.000
Agency	-0.79	0.05	.000	-0.58	0.04	.000
Communion	-0.11	0.05	.030	-0.10	0.04	.012
Slope predicted by						
Gender	0.17	0.27	.524	0.29	0.26	.255
Father bond	0.02	0.02	.341	0.00	0.02	.812
Mother bond	0.03	0.02	.120	0.03	0.02	.060
Agency	0.03	0.02	.085	0.02	0.02	.138
Communion	-0.01	0.02	.750	-0.01	0.02	.529
Time varying effects general interpersonal distress						
Negative beliefs				0.36	0.08	.000
Positive beliefs				0.39	0.08	.000
Lack of cognitive confidence				0.38	0.07	.000
Need to control thoughts				0.49	0.09	.000
Cognitive self-consciousness				0.11	0.07	.104
Anxiety symptoms				0.26	0.07	.000
Depression symptoms				0.39	0.06	.000

On average, more sub-optimal parental bonds to father and mother were associated with higher levels of general interpersonal distress whereas lower scores on agency and communion were associated with higher levels of general interpersonal distress at the initial status in both models (see **Table 1**). Neither of the baseline time invariant covariates were significantly associated with the rate of change in general interpersonal distress in both models. The controlled effect of all dysfunctional metacognitive belief domains except cognitive self-consciousness predicted greater general interpersonal distress over time. Symptoms of anxiety and depression also predicted greater general interpersonal distress over time. The results indicate that fluctuations in dysfunctional metacognitive beliefs, anxiety and depression symptoms at each timepoint predict fluctuations in general interpersonal distress at that same time point over and above baseline covariates, thus explaining within-person variation and time-specific influences in general interpersonal distress over time.

## 4 Discussion

The current study aimed to investigate metacognitive belief domains as within-person predictors of interpersonal distress over time. In line with our hypotheses, we found that all factors except gender were associated with higher levels of interpersonal distress at baseline. Contrary to our hypotheses, perceived parental bonds and interpersonal style factors agency and communion did not predict the trajectory of interpersonal distress over time at the between-person level. Time-varying symptoms of anxiety and depression were associated to the development of interpersonal distress at the within-person level. Nonetheless, all metacognitive belief domains, except cognitive self-consciousness, made unique contributions at the within-person level predicting greater interpersonal distress whilst controlling for the baseline characteristics and within-person levels of anxiety and depression symptoms over time.

Parental bonds to both father and mother were significantly associated with interpersonal distress at baseline supporting them as relevant to interpersonal distress at the cross-sectional level as previously reported (e.g., 53; 54). However, between-person differences in perceived parental bonds were not significantly related to the trajectory of interpersonal distress over time. This stands in contrast to research demonstrating associations between negative parental bonds, social isolation, loneliness, and internalizing/externalizing mental disorders over time (17, 55). Similarly, lower levels of agency indicating a submissive and non-assertive style, in addition to lower levels of communion indicating a detached and cold style, significantly predicted levels of interpersonal distress at the cross-sectional level, which is in line with research showing that submissive and detached behaviours are related to interpersonal problems, interpersonal sensitivity and difficulties in regulating emotions (20, 56, 57). However, between-person differences in these styles at baseline did not predict the trajectory of interpersonal distress over time.

At the within-person level, both symptoms of anxiety and depression contributed to the development of interpersonal distress over time, above and beyond the included baseline covariates. This aligns with recent research showing that depression and interpersonal distress covaries at the within-person level such that increases in one domain is associated with the other over time (58) and findings linking interpersonal problems to anxiety longitudinally (59).

The finding that within-person variation in metacognitive beliefs (except for the cognitive self-consciousness domain) contributed to interpersonal distress, are in line with the metacognitive model (23) where biases in metacognition underly psychological dysfunction including general interpersonal distress. It also aligns with previous findings in non-clinical (26) and clinical samples (27, 28) and a recent study showing that the same metacognitive belief domains predicted personality functioning (including interpersonal functioning) beyond baseline maladaptive personality traits over time and at the within-person level (60).

The unique contribution of metacognitive beliefs in the current study on top of the included covariates are notable given that stable internal cognitive working models of parental bonding have been reported as stable and unaffected by for instance depressive symptomatology over several decades (61, 62), and more stable interpersonal styles have also been associated with interpersonal distress and psychopathology risk more broadly (e.g., 19). However, this finding is in line with the metacognitive model which postulates that cognition is dominated by the effects resulting from the MCS and dysfunctional self-regulation (i.e., the CAS) which ultimately leads to increases in both emotional and interpersonal distress (23).

Our results cannot directly address the direction of the relationship between metacognitive beliefs and interpersonal distress. However, they have implications for individual patient treatment and suggest that metacognitive change could be relevant to alleviate interpersonal distress, potentially irrespective of perceived parental bonds, interpersonal style configurations, and severity of emotional distress symptoms. This suggestion is in line with the key mechanisms of change targeted in MCT, focusing on modifying metacognitions and corresponding CAS strategies (25). We note that four out of five MCQ-30 subdomains emerged as independent predictors, indicating that there are unique contributions from different metacognitive belief domains to general interpersonal distress, suggesting that these links therefore should be evaluated and appropriately conceptualized and modified at an individual patient level. For example: Negative metacognitive beliefs (e.g., “worrying is uncontrollable”) might influence interpersonal distress as they prohibit disengagement of the CAS generally; beliefs about the need to control thoughts (e.g., “If I did not control a worrying thought, and then it happened, it would be my fault”) could result in excessive reassurance seeking to avoid feared consequences caused by thoughts. In line with these suggestions, MCT is found to be effective in alleviating interpersonal problems across common mental health disorders (including personality disorders), treatment formats and in diverse treatment settings (30, 63).

Future research could investigate whether the current findings are replicated during the course of treatment within-individuals and utilize cross-lagged panel models testing the directionality of influences between the included predictors, which would constitute a better mechanistic test of the proposed associations (64). Additional measures of interpersonal problems and distress could also be explored in relation to metacognitive beliefs and use of information from significant others could be especially relevant in researching effects for the individual and close others. Further, even though we controlled several relevant factors in investigating the relationships between metacognitive beliefs and interpersonal distress, future studies could control other hypothesized factors suggested to underly interpersonal problems and distress such as interview-based assessments of attachment styles or other interpersonal style domains (e.g., personality traits).

Several limitations should be acknowledged and considered in interpretation of the current findings. The slope factor should be interpreted as indexing average change across the study period rather than a universal developmental trajectory, and its magnitude should be interpreted cautiously given the age-diverse sample and potential repeated measurement effects. Future studies with *a priori* directional hypotheses and shorter time spans may benefit from complementary modeling approaches that explicitly test reciprocal temporal dynamics. The sample was gathered at convenience and all data relied on self-report which is relevant as self-other agreement on interpersonal measures tend to be moderate (e.g., 65). Findings may also not generalize to clinical samples. Attrition was substantial across the included time-points, but we used state-of-the-art FIML for handling missing data. Furthermore, even though this constitutes the first test of metacognitive beliefs on interpersonal distress over time within individuals, the analyses cannot speak to causal relationships or the directionality of influence between the predictors.

In conclusion, several dysfunctional metacognitive belief domains derived from the S-REF model emerged as unique predictors of interpersonal distress over time and within individuals above and beyond gender, perceived parental bonds to mother and father, interpersonal style factors of agency and communion, and within-person levels of anxiety and depression symptoms. Dysfunctional metacognitions could therefore be valuable targets in interventions seeking to alleviate interpersonal distress. MCT, which directly aims to modify biases in metacognition rather than trying to influence for example attachment/personality styles or the content of self-other-representations and relationships, could therefore be especially suitable for this purpose.

## Data Availability

The raw data supporting the conclusions of this article will be made available by the authors, without undue reservation.
