# Supplementary material for: Do dysfunctional metacognitive beliefs contribute to interpersonal distress beyond interpersonal styles, parental bonds, depression and anxiety? A prospective within-person study
Source: Front Psychiatry. 2026 Feb 24;17:1766358. doi: 10.3389/fpsyt.2026.1766358 (PMC12972750; doi:10.3389/fpsyt.2026.1766358)
Supplement: Supplementary file 1 [file Supplementaryfile1.docx]

**SUPPLEMENTARY MATERIALS**

**Frontiers in Psychiatry - Psychological Therapy and Psychosomatics**

| **Table S1: Number of missingness** | | | | | | | | | |
| --- | --- | --- | --- | --- | --- | --- | --- | --- | --- |
|  | | Valid | | Missing | | Mean | | Std. Deviation |  |
| pos_t1 |  | 1,413 |  | 5 |  | 9.416 |  | 3.386 |  |
| neg_t1 |  | 1,414 |  | 4 |  | 11.602 |  | 4.588 |  |
| cc_t1 |  | 1,413 |  | 5 |  | 11.151 |  | 4.709 |  |
| nc_t1 |  | 1,412 |  | 6 |  | 10.025 |  | 3.525 |  |
| csc_t1 |  | 1,413 |  | 5 |  | 13.018 |  | 4.238 |  |
| pos_t2 |  | 762 |  | 656 |  | 9.752 |  | 3.575 |  |
| neg_t2 |  | 763 |  | 655 |  | 11.285 |  | 4.466 |  |
| cc_t2 |  | 761 |  | 657 |  | 11.522 |  | 4.869 |  |
| nc_t2 |  | 761 |  | 657 |  | 9.770 |  | 3.561 |  |
| csc_t2 |  | 763 |  | 655 |  | 12.614 |  | 4.295 |  |
| pos_t3 |  | 648 |  | 770 |  | 9.801 |  | 3.802 |  |
| neg_t3 |  | 648 |  | 770 |  | 10.842 |  | 4.402 |  |
| cc_t3 |  | 648 |  | 770 |  | 11.553 |  | 5.036 |  |
| nc_t3 |  | 648 |  | 770 |  | 9.575 |  | 3.411 |  |
| csc_t3 |  | 648 |  | 770 |  | 12.295 |  | 4.451 |  |
| pos_t4 |  | 610 |  | 808 |  | 9.817 |  | 3.854 |  |
| neg_t4 |  | 610 |  | 808 |  | 10.878 |  | 4.552 |  |
| cc_t4 |  | 610 |  | 808 |  | 11.504 |  | 5.129 |  |
| nc_t4 |  | 610 |  | 808 |  | 9.591 |  | 3.535 |  |
| csc_t4 |  | 610 |  | 808 |  | 12.129 |  | 4.668 |  |
| gad_t1 |  | 1,415 |  | 3 |  | 7.342 |  | 5.197 |  |
| gad_t2 |  | 766 |  | 652 |  | 7.084 |  | 5.018 |  |
| gad_t3 |  | 650 |  | 768 |  | 6.977 |  | 4.991 |  |
| gad_t4 |  | 611 |  | 807 |  | 6.519 |  | 5.107 |  |
| phq_t1 |  | 1,415 |  | 3 |  | 9.272 |  | 6.407 |  |
| phq_t2 |  | 765 |  | 653 |  | 9.652 |  | 6.468 |  |
| phq_t3 |  | 648 |  | 770 |  | 9.747 |  | 6.573 |  |
| phq_t4 |  | 610 |  | 808 |  | 9.327 |  | 6.651 |  |
| pbif_t1 |  | 1,360 |  | 58 |  | 30.659 |  | 8.631 |  |
| pbim_t1 |  | 1,397 |  | 21 |  | 30.148 |  | 9.256 |  |
| agent1 |  | 1,413 |  | 5 |  | -7.601 |  | 8.553 |  |
| communt1 |  | 1,414 |  | 4 |  | 3.933 |  | 8.379 |  |
| POS = Positive beliefs about worry, NC = Need to control thoughts,  CC = Lack of cognitive confidence, CSC = Cognitive self-consciousness,  GAD = Anxiety symptoms, PHQ = Depression symptoms,  PBIF = Parental bond: Father, PBIM = Parental bond: Mother  AGEN = AGENCY, COMMUN = Communion | | | | | | | | | |

| **Table S2:** Differences in initial levels between completers and non-completers | | | | |
| --- | --- | --- | --- | --- |
|  | | ***M(SD)*** | ***t*** | ***p-value*** |
| Positive metacognitive beliefs | |  |  |  |
|  | Completers | 9.34 (3.39) | .774 | .220 |
|  | Non-completers | 9.48 (3.39) |  |  |
| Negative metacognitive beliefs | |  |  |  |
|  | Completers | 11.48 (4.58) | .835 | .404 |
|  | Non-completers | 11.69 (4.59) |  |  |
| Lack of cognitive confidence, | |  |  |  |
|  | Completers | 11.43 (4.85) | -1.911 | .056 |
|  | Non-completers | 10.94 (4.59) |  |  |
| Need to control thoughts | |  |  |  |
|  | Completers | 10.04 (3.51) | -.137 | .891 |
|  | Non-completers | 10.01 (3.54) |  |  |
| Cognitive self-consciousness | |  |  |  |
|  | Completers | 12.98 (4.30) | .326 | .744 |
|  | Non-completers | 13.05 (4.19) |  |  |
| Anxiety symptoms | |  |  |  |
|  | Completers | 7.33 (5.23) | .090 | .928 |
|  | Non-completers | 7.35 (5.17) |  |  |
| Depression symptoms | |  |  |  |
|  | Completers | 9.75 (6.74) | -2.438 | .015 |
|  | Non-completers | 8.91 (6.12) |  |  |
| Age | |  |  |  |
|  | Completers | 31.52 (12.98) | -4.99 | .000 |
|  | Non-completers | 28.40 (10.37) |  |  |
| Parental bond: Father | |  |  |  |
|  | Completers | 30.83 (8.65) | -0.622 | .267 |
|  | Non-completers | 30.53(8.62) |  |  |
| Parental bond: Mother | |  |  |  |
|  | Completers | 30.64 (6.74) | -1.75 | .080 |
|  | Non-completers | 29.77 (6.12) |  |  |
| Agency | |  |  |  |
|  | Completers | -8.71 (8.90) | 4.29 | .000 |
|  | Non-completers | -6.76 (8.19) |  |  |
| Communion | |  |  |  |
|  | Completers | 3.65 (8.53) | 1.08 | .138 |
|  | Non-completers | 4.14 (8.26) |  |  |

**Analysis of missing data patterns**

Incomplete data patterns can be represented as the proportion of data or coverage for each covariance of scores between two variables. At any time, the highest available data was 99% of the participants and the lowest coverage was 35%. As attrition is a common occurrence in longitudinal data, we consider that the pattern of data loss is not too great (Tables S1).

| **Table S3:** Covariance coverage across time | | | | | | | | |  |
| --- | --- | --- | --- | --- | --- | --- | --- | --- | --- |
|  |  | 1 | 2 | 3 | 4 | 5 | 6 | 7 |  |
| 1 | General interpersonal distress_T1 | .99 |  |  |  |  |  |  |  |
| 2 | General interpersonal distress _T2 | .54 | .54 |  |  |  |  |  |  |
| 3 | General interpersonal distress _T3 | .46 | .40 | .46 |  |  |  |  |  |
| 4 | General interpersonal distress _T4 | .43 | .37 | .35 | .43 |  |  |  |  |

We investigated whether any differences exist between completers and non-completers at T2 – T4, using the scores at T1 on general interpersonal distress. Although attrition was not systematically related to interpersonal distress at earlier timepoints, by the final measurement completers reported slightly higher distress than non-completers. This suggests that participants experiencing greater distress may have been more likely to remain engaged, which could have implications for the generalizability of findings at later waves. One possible explanation is that individuals experiencing greater distress were more motivated to remain engaged in the study. Conversely, those with lower distress may have seen less personal relevance and dropped out.

| **Table S4:** Differences in initial levels between completers and non-completers | | | | |
| --- | --- | --- | --- | --- |
| **Time** | | ***M(SD)*** | ***t*** | ***p-value*** |
| **General interpersonal distress** | |  |  |  |
| Time 2 | |  |  |  |
|  | Completers | 37.84 (17.99) | -0.92 | .178 |
|  | Non-completers | 36.96 (17.90) |  |  |
| Time 3 | |  |  |  |
|  | Completers | 38.21(17.89) | -1.49 | .067 |
|  | Non-completers | 36.78 (18.00) |  |  |
| Time 4 | |  |  |  |
|  | Completers | 38.41 (18.52) | -1.78 | 039 |
|  | Non-completers | 36.70 (17.48) |  |  |

We used logistic regression to estimate the extent to which variables in previous times (i.e., T1 – T3) predict attrition from subsequent times (T2 – T4). If variables in the analysis model are related to attrition, it is unlikely that dropout occurred completely at random (i.e., resulting in data that are missing completely at random [MCAR]). The results mainly indicated that the logistic regression models were not significant in the extent to which variables in previous times predict attrition from subsequent ones.

| **Table S5:** Logistic regression for whole time missing data analyses | | | | | | |
| --- | --- | --- | --- | --- | --- | --- |
|  | **Time 2** | | **Time 3** | | **Time 4** | |
|  | B (SE) | *p*-value | B (SE) | *p*-value | B (SE) | *p*-value |
| General interpersonal distress_T1 | 0.00 (0.00) | .356 |  |  |  |  |
| General interpersonal distress _T2 |  |  | 0.01 (0.00) | .291 |  |  |
| General interpersonal distress _T3 |  |  |  |  | 0.00 (0.00) | .701 |

**Figure S1**: Observed individual trajectory plots for a random subsample in the completers (*n* = 50)
